# Strategies to improve research capacity across European general practice: The views of members of EGPRN and Wonca Europe

**DOI:** 10.1080/13814788.2018.1546282

**Published:** 2019-01-04

**Authors:** Caroline Huas, Davorina Petek, Esperanza Diaz, Miquel A. Muñoz-Perez, Peter Torzsa, Claire Collins

**Affiliations:** a UVSQ, CESP, INSERM, Université Paris-Saclay, Univ. Paris-Sud, Villejuif, France;; b Fondation santé des étudiants de France, Paris, France;; c Department of Family Medicine, Faculty of Medicine, University of Ljubljana, Ljubljana, Slovenia;; d Executive Board, European General Practice Research Network, Maastricht, The Netherlands;; e Department of Global Public Health and Primary Care, University of Bergen, Bergen, Norway;; f Norwegian Centre for Migration and Minority Health, Oslo, Norway;; g Departement de Salut, Generalitat de Catalunya, Institut Català de la Salut. IDIAP-Jordi Gol, Barcelona, Spain;; h Department of Family Medicine, Semmelweis University Faculty of Medicine, Budapest, Hungary;; i The Irish College of General Practitioners, Dublin, Ireland

**Keywords:** General practice, research, capacity, family medicine, network

## Abstract

**Background:** The effectiveness of any national healthcare system is highly correlated with the strength of primary care within that system. A strong research basis is essential for a firm and vibrant primary care system. General practitioners (GPs) are at the centre of most primary care systems.

**Objectives:** To inform on actions required to increase research capacity in general practice, particularly in low capacity countries, we collected information from the members of the European General Practice Research Network (EGPRN) and the European World Organization of Family Doctors (Wonca).

**Methods:** A qualitative design including eight semi-structured interviews and two discursive workshops were undertaken with members of EGPRN and Wonca Europe. Appreciative inquiry methods were utilized. Krueger’s (1994) framework analysis approach was used to analyse the data.

**Results:** Research performance in general practice requires improvements in the following areas: visibility of research; knowledge acquisition; mentoring and exchange; networking and research networks; collaboration with industry, authorities and other stakeholders. Research capacity building (RCB) strategies need to be both flexible and financially supported. Leadership and collaboration are crucial.

**Conclusion:** Members of the GP research community see the clear need for both national and international primary care research networks to facilitate appropriate RCB interventions. These interventions should be multifaceted, responding to needs at different levels and tailored to the context where they are to be implemented.

KEY MESSAGES

- There is a mutual benefit from interactions between countries at different stages of research capacity building.
- An internationally constructed general practice research network aimed at assisting countries to develop their research capacity is required.
- Research capacity is a complex activity and involves individual, institutional and environmental aspects.

## Background

The effectiveness of any national healthcare system is strongly correlated with the strength and position of primary care within that system [1]. General practitioners (GPs) are central to the primary care system in most European countries and they have an integral role in assessing the health needs of the population. A strong research basis is essential for a firm and vibrant primary care system [2,3]. Research in general practice is a relatively young discipline. Moreover, the status/recognition of general practice and general practice research across Europe differs widely. The implementation of research in general practice and its homogenization across Europe is not straightforward.

The World Organization of National Colleges, Academies and Academic Associations of General Practitioners/Family Physicians (Wonca) is the organization of family doctors—it has both a European and a World network. The European General Practice Research Network (EGPRN) is an organization of GPs and other health professionals involved in research in primary care and general practice/family medicine. The focus of this paper is on the views of EGPRN and Wonca Europe members regarding the actions required to increase research capacity in general practice in Europe, particularly in low capacity countries. Wonca Europe and EGPRN have been very proactive in contributing to the formation and development of research across Europe (and further afield) [4,5].

In 2009, the EGPRN published a research strategy for Europe highlighting specific priorities for future focus; it also outlined key actions required to build research capacity in low capacity countries [6]. However, nearly 10 years on, disparities within Europe in terms of research capacity persist and are expounded in the literature (Table 1 and Table 2).

**Table 1.** Summary of key barriers to research.

Low research capacity [29]
Lack of time [15] Lack of connection to skilled research [15]. Lack of research expertise or access to means to improve it [15,29]
Strong publication as the main outcome [16]
Lack of networking opportunities for mentoring and career development [9]
GPs do not have the time or patience for process (e.g. ethics application); their success depends on meeting patients’ needs [16,29]
Collaborative priority settings between researchers, and end users of research is more time-consuming than traditional approaches to project development [8]

**Table 2.** Summary of key facilitators for research.

Fair relationships between academic and practice partners are important [20]
Financial support for practicing clinicians to conduct research [30]
Access to senior researchers [16]
Timeliness of the research itself [16]
Personal contact among major stakeholders [16]
‘Knowledge brokers’ might be required to translate the research into actionable, policy relevant messages [23]
The quality of the research undertaken [16,19]
Research that supports existing policy directions [5,16]
Peer support and a formal academic posting for clinicians engaged in research [16]
‘Simplicity over complexity’ (for the focus of research) [16]
Practice relevant activities that promote research education, dissemination and collaboration partnership opportunities [17]
Appropriate infrastructure, national research institutes and university departments of family medicine [5,17]
Leadership [17]
Mechanisms for sustainability ‘within healthcare organization’ [17], research practice based networks [30]
The use of pre-existing historical partnerships with ongoing dialogue [8]
Length of the programme providing stability and long-term relationships [8]
Peer-reviewed journal(s) [29]

Research capacity building (RCB) can be defined as ‘a process of individual and institutional development which leads to higher levels of skills and greater ability to perform useful research’ [7]. Building research capacity in health services is essential to produce a sound evidence base for decision-making in policy and practice. RCB can be conceptualized as three integrated and interrelated levels: individual, organizational and environmental [8]. The three levels overlap substantially and there are no clear boundaries between them [9]. To be successful and sustainable, RCB interventions should take place at multiple levels, be context-specific, and dynamic [10].

This work was undertaken while preparing a proposal for a Horizon 2020 research grant call aimed at increasing research capacity in low capacity countries [11]. The key objective of the research reported here was to explore possible strategies for bridging the divide in general practice research in Europe.

## Method

In this research, a qualitative design was employed using workshops and individual interviews, to explore possible strategies for bridging the divide in general practice research in Europe. These two methods provided cross verification through triangulation. They enabled richness of ideas from several viewpoints of researchers, academics, GPs and leaders of different settings from low and high capacity countries. Furthermore, this was a bottom-up organizational approach, an accepted approach to capacity building in health [12]. Data collection continued until data saturation was reached.

### Ethics

The study followed the principles of the Declaration of Helsinki.

### Workshops

Two workshops were held in May 2016 (EGPRN, *n* = 35 participants, 27 countries) and June 2016 (Wonca Europe, *n* = 29 participants, 13 countries) with representatives from both high and low capacity countries. It was considered that the interactions between GPs and researchers of varying experience and from countries at different developmental stages in terms of RCB would be edifying. Note-takers recorded these discussions and assigned comments according to the individual’s country.

### Interviews

A series of eight one-to-one interviews, with EGRPN members from eight countries were held with key informants. A semi-guided interview was utilized. Purposive sampling was used to select participants from different European countries. In addition to a balance between participants from low and high capacity countries (low capacity countries are below 70% of the EU average on the Composite Indicator of Research Excellence as defined by the EU) [11], we aimed for variety of age and academic position. The interviews lasted from seven to 26 min with the average time of 17.5 min. The interviews were audio recorded and transcribed in full. Only the country of each interview participant is reported here for consistency with reporting for the workshop participants but also to protect the individual’s identity. Questions were designed according to best practice for priority setting [8].

### Analysis

Appreciative inquiry methods were utilized both in workshops and interviews to inform changes in research participation. Appreciative inquiry is an organizational development process or philosophy that engages individuals within an organizational system in its renewal and promotes a positive focus on problem-solving [13].

Open coding of the transcripts of both workshops and interviews was undertaken and themes were deduced using open coding techniques. Two researchers (DP, CC) performed coding and the differences in coding were resolved by discussion. Sub-themes were sought to provide a full view of the participants’ opinions [14]. ‘Participants’ refers to both workshop and interview participants.

## Results

The themes, which were identified in workshops and interviews, categorized into barriers, solutions and outcomes are summarized in Table 3 and are classified at an individual, organizational and environmental level. These are combined and presented under the five main overarching strategies suggested in our findings to improve RCB in general practice across Europe. It was recognized that the initial problem for low capacity countries is the establishment of research topics locally and the acquisition of diverse research skills, while building international cooperation at the same time. Hence, several strategies are required simultaneously, which incorporate more than knowledge acquisition. Table 3 also summarizes the solutions and potential outcomes suggested by participants.

**Table 3.** Identified barriers, suggested solutions and their outcomes.

Identified barriers	Suggested solutions	Outcomes
***Individual***		
Lack of human resources	Availability of mentors	Mentorships
No individual research posts	Peer-learning	National and international masterclasses
No mentorship	Improve soft skills	
No leadership	Research competences, and specific knowledge on general practice research	Grants for visits and protected time
Brain drain		
		Process learning courses (face-to-face, e-learning, etc.)
Low access to literature, courses, conferences, etc.		
Few research contacts	Participation in international projects	Peer-learning
Financial difficulties		
		Database
		Collaborative projects
***Organizational***		
Low knowledge translation	Improve mentorship	Train the trainers
Lack of certification	Rewarded mentorship	Visits from high performing countries
No clinical networks	Increase environmental face	Overheads for receiving institution
Low involvement/influx with own organization		
		Accreditation
		Database
		Access to literature
		Process learning
***Environmental***		
Low cooperation stakeholders	Improve mentorship and leadership	Courses (face-to-face, e-learning, etc.)
No appropriate leadership	Improve knowledge translation	
Low collaboration with high performing countries	Improve external contacts/environmental face	Peer-learning
		Database
Low visibility	Increase visibility in national and European context	Collaborative projects
		Environmental face advances
		Process learning
		Application to EU funding

### Improved visibility of primary care research in low capacity countries

The participants mentioned several mechanisms that would make low capacity countries more visible on the map of EU primary care research: the establishment of formal institutions (institutes or academic departments of family medicine (FM), ‘academic pools’) and the creation of sustainable platforms for research within existing networks and organizations (EGPRN, SIG, UEMO) were called for. National strategies and budget lines would be required. Protected time, along with the acquisition of human resources (PhD, mentors, experienced and trusted teachers and researchers) and establishing a career pathway for GP researchers were noted as necessary to develop individuals and prevent ‘brain drain’.

‘We would need protected time for research because most researchers are at the same time working as GPs and teachers on various levels’. (Slovenia)

‘One option would be trying to arrange meetings with regulatory entities and expose them to the advantages of research in general practice and to give examples of other successful countries’. (Portugal)

### Knowledge Acquisition

A wide range of knowledge acquisition needs were mentioned to improve research in low capacity countries—classical courses, ongoing career support structures and utilizing information and communications technology (ICT) and online facilities to identify and link domain experts and learning opportunities were suggested. Established classical research courses should be adapted for local needs, including local teachers, and accounting for regional aspects like the healthcare system characteristics. An adequate curriculum for the courses and learning options must be developed. Also, research posts and career support after formal completion of education were needed, together with the improvement of ‘soft skills’, such as project management, and writing project proposals.

‘… learning should also include work in small groups, mentoring. Good basic material is needed for further training, gives common ground. There is some stuff available but also a gap in this material’. (Belgium)

Certification of educational programmes and learning activities were also mentioned. On the European level, there is a need for a system that can link with the accreditation institutions in each country. Nationally, a system of recognition and certification, given by medical associations or the Ministry of Health in each country is required both for teachers and students according to the participants.

### Mentoring and exchange possibilities

Participants both from high and low capacity countries not only recognized the importance of established personal and institutional contacts from previous research collaboration, but also of new contacts and connections between the research institutions and human resources. 

According to participants, the development of good mentors is a process that needs adequate and sufficient timing. The establishment of a database with a coordination point for mentors and researchers was mentioned. Such a database would provide the possibility of finding colleagues to work with and mentors according to one’s activities, knowledge, and interests.

‘We would like to get the possibilities to send young researchers to other research institutions to improve their skills and cooperate on various research projects’. (Slovenia)

Exchange programmes specifically for research, visiting academics from the high to the low capacity countries, opportunities for participation at international conferences and access to research literature were specifically mentioned. Financial resources were highlighted as key for any visits, collaboration and exchange programmes.

### Networking and research networks

Networking was mentioned at individual level as an opportunity and at the organizational level in terms of structure and support.

‘Currently no clinical research networks are based around specific topics’. (Ukraine)

As opposed to a vision of high capacity countries having no needs and all the benefit being to the low capacity countries, the benefit for both parties was emphasized: reflective process of joint work, integration of other disciplines, sharing experience, and development of good practices were considered in this regard.

A clinical network of practices in each country was considered an important aspect of RCB. The practices are the primary source of data collection and represent the final place for the implementation of many research outcomes in primary care. Participants mentioned several preconditions for the development of national research networks with quality data output and highlighted the significant role of the local level.

‘Learn how to start the development of clinical networks, it is a developing process, should be linked to other projects, exercise it locally. Link clinical networks with other networks, there is quality assurance’. (Belgium)

### Collaboration and communication with industry, authorities and other stakeholders

Collaboration and communication to highlight the importance of primary care research and its potential contribution to the evidence base and to changes in care were noted as required but often overlooked. Generating public interest in primary care research was considered especially important. A culture of working with other stakeholders must be developed, according to the participants. Several aspects of possible collaboration with the pharmaceutical industry and different stakeholders were mentioned, for example, awareness of possible manipulation, respect for ethical principles and culture and experience of working with other stakeholders.

To establish cooperation with small enterprises, industry, other public institutions, an overview of enterprises in the country was mentioned as needed along with a communications strategy and a policy on how to develop networks with enterprises and agencies. Political support was also noted as a requirement.

‘Development of a structured European map for primary healthcare systems to facilitate generalization of results from research studies’. (Greece)

## Discussion

### Main findings

Several overarching themes appeared which can be linked to the individual, organizational, and environmental levels of RCB. At the individual level, research training is necessary along with mentorship, peer-learning and protected time. At the organizational level, local clinical networks, certification of learning activities, and support structures are required. At the environmental level, increased visibility, formal research institutions, national strategies, dedicated budget lines, and communication with a wide range of stakeholders will increase the impact of primary care research.

International cooperation, through collaborative research projects, would be effective to provide research skills to the low capacity countries and to create international networks.

One of the main actions would be the establishment of mentoring and exchange programmes.

### Interpretation of findings in the context of existing evidence

The identified literature supports some of the challenges and barriers highlighted in this research, in terms of the implementation of interventions for RCB in primary care. Among the previously identified challenges are the lack of protected time, the limited research-related human resource capacity to mentor novice researchers [15–17], the issues of sustaining communication, a range of networking and capacity-enhancement needs, and balancing the demands to foster research excellence with the needs to create infrastructure and advocate for adequate research funding [18]. Collaboration with stakeholders is crucial for the success of interventions to improve research capacity [16]. These relationships include those between academic and practice partners, across disciplines [19], with health policy makers [20,21], patients [22] and the pharmaceutical industry. ‘Knowledge brokers’ might sometimes be required to translate the research into actionable, policy-relevant messages [23].

RCB in countries without an organizational infrastructure is challenging, and it needs to be flexible and tailored to the context [9]. One central question is how to empower individual researchers to build enough relevant high-quality science and become central in their national practice-based network to enhance the necessary infrastructural development [5,17,18,24].

In a globalized society, networking with international peers might help to build confidence and the capacity of researchers, supporting the quality of the research [5]. Collaboration is seen as mutually useful and has to be appreciated as a reflective and equitable process of joint learning [9,25]. Our results corroborated this approach and reinforced the idea of mutual benefit to be gained from the interactions between countries at different stages and with varying research capacity. The importance of training, and particularly learning by doing was emphasized as explored elsewhere [26,27].

### Limitations

Workshop participants were self-selected, however, as both low and high capacity countries were included and all were family doctors with research awareness and experience, selection bias in terms of not being representative can be considered low. The interview participants were sampled purposively to ensure a mix across countries, age, gender and research experience, reducing this potential bias. Furthermore, the qualitative design and the use of workshops and interviews led to data saturation.

### Implications for further research

A key implication is the need to establish an internationally constructed general practice research network that helps each country to develop its own programme supported by the international community. It is recognized that this is not the responsibility of any one organization and that resources are required to achieve this. However, the EGPRN could be a natural main driver of this. The impact of this network needs to be measured and evaluated. However, RCB projects are often complex and hard to evaluate, with a necessity to assess the effects of the different interventions at the individual, institutional and environmental levels. A multi-objective composite set of measures of research performance that captures different types of outputs is suggested as the optimal way to determine the success of such a programme [16]. Furthermore, as RCB is a process, different outcome measures may be required at different stages of the intervention [28,29].

The evaluation of the achievements of RCB is not a simple task; it has to be adapted to the different stages of RCB and different types of output [6]. Innovative evaluation techniques and measures should be researched and tested particularly for RCB activities at the organizational and environmental levels.

## Conclusion

Members of the general practice research community feel the clear need both for national and international research networks to facilitate appropriate RCB interventions [30]. These interventions should be multifaceted, responding to needs at different levels and tailored to the context where they are going to be implemented. RCB strategies need to be flexible and financially supported [30]. Leadership, ongoing dialogue and collaboration are crucial.
